# Direct and indirect effects of land‐use intensification on ant communities in temperate grasslands

**DOI:** 10.1002/ece3.5030

**Published:** 2019-03-05

**Authors:** Lisa Heuss, Michael E. Grevé, Deborah Schäfer, Verena Busch, Heike Feldhaar

**Affiliations:** ^1^ Animal Population Ecology, Animal Ecology I, Bayreuth Center of Ecology and Environmental Research (BayCEER) University of Bayreuth Bayreuth Germany; ^2^ Institute of Plant Sciences University of Bern Bern Switzerland; ^3^ Institute for Landscape Ecology Münster University Münster Germany

**Keywords:** arthropods, Formicidae, grassland management, grazing, mowing, species homogenization

## Abstract

Land‐use intensification is a major driver of local species extinction and homogenization. Temperate grasslands, managed at low intensities over centuries harbored a high species diversity, which is increasingly threatened by the management intensification over the last decades. This includes key taxa like ants. However, the underlying mechanisms leading to a decrease in ant abundance and species richness as well as changes in functional community composition are not well understood. We sampled ants on 110 grassland plots in three regions in Germany. The sampled grasslands are used as meadows or pastures, being mown, grazed or fertilized at different intensities. We analyzed the effect of the different aspects of land use on ant species richness, functional trait spaces, and community composition by using a multimodel inference approach and structural equation models. Overall, we found 31 ant species belonging to 8 genera, mostly open habitat specialists. Ant species richness, functional trait space of communities, and abundance of nests decreased with increasing land‐use intensity. The land‐use practice most harmful to ants was mowing, followed by heavy grazing by cattle. Fertilization did not strongly affect ant species richness. Grazing by sheep increased the ant species richness. The effect of mowing differed between species and was strongly negative for *Formica* species while *Myrmica* and common *Lasius* species were less affected. Rare species occurred mainly in plots managed at low intensity. Our results show that mowing less often or later in the season would retain a higher ant species richness—similarly to most other grassland taxa. The transformation from (sheep) pastures to intensively managed meadows and especially mowing directly affects ants via the destruction of nests and indirectly via loss of grassland heterogeneity (reduced plant species richness) and increased soil moisture by shading of fast‐growing plant species.

## 1 INTRODUCTION

In temperate Europe, grasslands have been extensively managed over centuries by grazing and low‐intensity mowing. These semi‐natural grassland ecosystems show a very high plant and animal diversity (Chytrý et al., 2015; Hejcman, Hejcmanová, Pavlů, & Beneš, 2013), but are threatened by increasing management intensity (or abandonment) over the last decades. A negative influence of increasing land use on species richness and abundance has already been documented for plants (Haddad, Haarstad, & Tilman, 2000; Harpole & Tilman, 2007; Kleijn et al., 2009; Socher et al., 2012), different arthropod groups (Allan et al., 2014; Chisté et al., 2016; Hendrickx et al., 2007; Simons et al., 2014), and on the overall functional diversity of communities (Birkhofer, Smith, Weisser, Wolters, & Gossner, 2015; Blüthgen et al., 2016; Flynn et al., 2009).

In Germany, about 28% of the agricultural land is used intensively as grassland, either as meadows or pastures (Statistisches Bundesamt, 2016). The three management practices used in agricultural grasslands are mowing, fertilization, and grazing (mostly by cattle and sheep). They differ in their effects on biodiversity in grasslands (Simons et al., 2014; Socher et al., 2012), and effects change along the management intensity gradient. Fertilization and mowing are often highly correlated (Blüthgen et al., 2012), and high intensities of both result in the homogenous vegetation structures and reduced plant diversity (Harpole & Tilman, 2007). This leads to overall multitrophic homogenization (Gossner et al., 2016) and therefore a decline in arthropod species richness and abundance (Haddad et al., 2009). Besides these indirect effects, mowing also has direct negative effects on arthropods (Marini, Fontana, Scotton, & Klimek, 2007; Nickel & Hildebrandt, 2003; Simons et al., 2017; Socher et al., 2012) as they are killed by machines (Thorbek & Bilde, 2004). In contrast, grazing affects the biotic environment by simplifying and thinning out vegetation and litter, which can, at a moderate level, increase plant diversity (Stewart & Pullin, 2008), an effect, that can vary between the different types of livestock (Socher et al., 2013). Thus, moderate grazing has a far less detrimental effect on arthropods than mowing and fertilization.

Ants are a key taxon in grasslands (reviewed in Wills & Landis, 2018). They are moderately diverse and can be highly abundant, making them important consumers and ecosystem engineers (Folgarait, 1998; Del Toro, Ribbons, & Pelini, 2012). Ants alter plant communities through predation of herbivorous insects (Sanders & van Veen, 2011), seed dispersal (Howe & Smallwood, 1982), and seed consumption (Dauber, Rommeler, & Wolters, 2006). Additionally, they build subterranean nests and thereby modify the soil structure, which increases plant diversity (Nemec, 2014; Del Toro et al., 2012) due to the creation of small islands for less competitive plant species, which would otherwise be outcompeted (Dean, Milton, & Klotz, 1997).

Land‐use intensification may negatively affect ant species richness (Dauber & Wolters, 2004, 2005), abundance, and colony density (Diaz, 1991; Folgarait, 1998) and functional community composition (Dauber & Wolters, 2005) in temperate grassland both directly and indirectly. However, the underlying mechanisms leading to the decrease in ant species richness, abundance, and the effects on species with distinct functional traits and therefore functional community composition are currently not well understood. The impact of the different management practices such as mowing, fertilization, and grazing have not yet been elucidated.

*Mowing* affects the entire grassland patch and can have a direct negative effect on ant species building nest mounds, as especially the aboveground parts of the nest are destroyed during mowing. Ant species which differ in their trophic niche and foraging behavior (Blüthgen & Feldhaar, 2010) might be affected differently by land use as well. Indirect effects of mowing on ant communities are the alteration of available food sources and changes of microhabitats. Thus, mowing instantly reduces the availability of resources associated with plant parts that are cut, such as floral and extrafloral nectar, as well as the abundance of arthropods and therewith potential prey items and trophobionts may be lacking (Socher et al., 2012). To date, studies on mowing effects on ants are, to our knowledge, mainly elucidating effects of low‐intensity mowing (max. two cuts per year). Pech, Dolanský, Hrdlička, and Lepš (2015), Noordijk et al. (2010), and Pérez‐Sánchez, Zopf, Klimek, and Dauber (2017) found that different mowing regimes led to highly site‐dependent responses of ants.

Direct effects of *fertilization* might be the disturbance of nesting sites by heavy machinery. However, the main effect of fertilization will be indirect. Pihlgren, Lenoir, and Dahms (2010) found fewer ant species in fertilized than in nonfertilized meadows and linked this to the enhanced growth of taller plant species. Such plant species reduce sun exposure and thus soil temperature, which can reduce the occurrence of ant species specialized to open habitats.

For *grazing*, positive (Bromham, Cardillo, Bennett, & Elgar, 1999), negative (Boulton, Davies, & Ward, 2005), and neutral (Bestelmeyer & Wiens, 2001; Whitford, Zee, Nash, Smith, & Herrick, 1999) effects have been found. However, grazing effects are to date not sufficiently studied in temperate grasslands with long grazing history (Pihlgren et al., 2010). Besides, it is poorly understood whether grazing affects ants directly due to the destruction of nesting sites by trampling of livestock or indirectly, through increased structural heterogeneity due to selectively removed plant biomass, localized fertilization by feces deposition, altered plant species richness, and reduced plant cover, which increases ground temperature (Boomsma & Van Loon, 1982).

In this study, we aim at disentangling the effects of the compound land‐use intensity (LUI; Blüthgen et al., 2012) and its three main components, grazing, fertilization, and mowing, on ant species richness and abundance in temperate grasslands. We analyze the importance of both direct and indirect effects of land‐use intensification and management type in temperate grasslands on ant community composition. We test whether the ant community composition changes along a land‐use intensity gradient and investigate which morphological and life history functional traits are reduced in communities when certain species disappear.

## 2 METHODS

### 2.1 Study site

This study was performed within the framework of the Biodiversity Exploratories, which includes three study sites in Germany, the Schwäbische Alb, Hainich‐Dün, and Schorfheide (Fischer et al., 2010) (www.biodiversity-exploratories.de). The three study regions reflect a gradient of increasing altitude and precipitation and decreasing annual man temperatures from northeastern to southwestern Germany (for detailed description of the regions see appendix). Each region (henceforth Alb, Hainich, and Schorfheide) comprises 50, relatively evenly distributed grassland plots of 50 m × 50m for comparative biodiversity studies along a land‐use gradient. For additional details, see Fischer et al. (2010).

### 2.2 Land‐use intensity index

The sampled grasslands are managed by local farmers as meadows, pastures, or mown pastures (mown and grazed by livestock) at different intensities. Land‐use intensities range from extensively used pastures to heavily fertilized meadows which are mown up to four times a year. Standardized interviews have been conducted every year to record the intensity and type of land use for every plot, considering *mowing intensity* (M *), fertilization intensity* (F), and *grazing intensity* (G). Mowing is measured as the number of cuts per year (ranging from zero to three cuts per year). Fertilization intensity is measured as the amount of nitrogen (in kg) applied per hectare and year from chemical fertilizer, manure, or slurry. Grazing intensity includes information on livestock type (sheep, cattle, and/or horses), number of livestock units, and duration of the grazing period (in days). All three land‐use components are integrated into the globally standardized land‐use intensity measure LUI by averaging the three measures after standardizing to a common scale:

$$\text{LUI}\left(i\right)=\sqrt{\frac{M\left(i\right)}{M\text{mean}}+\frac{F\left(i\right)}{F\text{mean}}+\frac{G\left(i\right)}{G\text{mean}}}$$

where *M*mean, *F*mean, *G*mean are mean values for all plots of each region (Blüthgen et al., 2012). To quantify long‐term land‐use intensity, a mean LUI for the years 2011 to 2015 was calculated.

### 2.3 Sampling design

Due to the presence of livestock or mowing activities, we conducted the sampling on 110 of the 150 plots (Table 1). Per 50 m × 50 m plot, we combined the three different sampling methods of pitfall trapping, hand sampling, and baiting in order to achieve a robust representation of ant species richness. Sampling was conducted in transects along all four edges of each plot, with transects of each edge being 50 m long and 2 m wide. In total, 12 pitfall traps were placed per plot along the transects (for detailed description see appendix). In 2014, we sampled pitfall traps in the regions Alb (June) and Hainich (August) and July 2015 in Schorfheide. In 2015 (Alb in May, Hainich in June, and Schorfheide in July), we walked the two‐meter wide transects, collected all ants visible foraging on the ground or in vegetation. All visible ant nests were counted and sampled once on the same transects through walking along all four edges and searching for ant nests on the surface, as a measurement for ant abundance. In addition, 16 bait stations were placed along the edges (four per edge at a distance of 7.5, 17.5, 32.5, and 42.5 m from the corners of the plots). Bait stations were installed and monitored for one hour and contained artificial diet with different protein to carbohydrate ratios (for a detailed description see Supporting information Appendix S1). After collection, all ants were stored in ethanol to conserve them for further analysis. Ants were identified using Czechowski, Radchenko, Czechowska, and Vepsäläinen (2012), Radchenko and Elmes (2010), Seifert (2007), Seifert and Galkowski (2016), Seifert and Schultz (2009).

**Table 1:** Overview over the three study regions. Stated is the number of sampled plots, range and mean number of ant species found, range of land‐use intensity (LUI), and number of plots with different types of livestock

Region	Plots	Ant species Min–Max Mean (*SD*)	LUI Min–Max Mean (*SD*)	Livestock			
				None	Cattle	Sheep	Cattle & horses
Alb	37	0–14 5.32 (*SD* 4.31)	0.46–3.11 1.63 (*SD* 0.73)	17	2	16	2
Hainich	33	0–15 6.39 (*SD* 3.65)	0.65–3.05 1.52 (*SD* 0.64)	5	13	15	0
Schorfheide	40	0–9 3.77 (*SD* 2.09)	0.98–2.63 1.57 (*SD* 0.35)	17	23	0	0

Notes

Plots with no livestock are managed as meadows (mown and fertilized at different intensities).

### 2.4 Environmental variables

Biotic variables—Vegetation was sampled in 2015 from May to June. In a representative subplot of 4 m × 4 m, all vascular plant species were recorded, average vegetation height was measured, and the *coverage of herbs*, *shrubs*, *bryophytes,* and *litter* was estimated in percent. Aboveground community biomass (gram/m^2^) was sampled at the same time by cutting the vegetation at a height of 2‒3 cm in four 0.5 × 0.5 m subplots, dried, and weighed.

Abiotic variables—Each plot was equipped with a meteorological station measuring *ground temperature* (°C) at ten centimeters above the ground and s*oil moisture* measured in percentage of volumetric water content at ten centimeters depth. Temperature and moisture were measured continuously from May to August in 2014 and 2015 and then averaged over the whole period.

### 2.5 Ant traits

We measured morphological traits for each species occurring on each plot under the binocular (Leica M165 C binocular system and the software “Leica Application Suite”). We measured up to ten individuals from different plots for each species and used trait means for the analyses. We measured the following traits: *Weber's length* (mesosoma length), *pronotum width*, *head length* and *width*, *femur*, and *tibia length* of the hind leg as well as *eye width*. We chose and measured traits as suggested by Parr et al. (2017) and references therein. For all traits, we calculated the relative values by dividing them by Weber''s length and used Weber's length as absolute values.

We extracted life history traits of all ant species mostly from Arnan, Cerdá, and Retana (2017) and partly from Seifert (2007, 2017). As traits, we used *behavioral dominance*, *number of queens per nest*, *number of nests*, *colony size,* and assumed *nutritional niche* (values for the latter are based mostly on expert knowledge by Seifert (2017), but also based on former published work). Further, we used the *foraging strata*, calculated from values a specific ant species is most likely found foraging on assumed by Seifert (2017). As a measurement of the ants' size, we used the mean *Webers’ length* per species. The trait data and a more detailed description of the trait categories are provided in the Supporting information Appendix: Tables S2, S3, and S4.

### 2.6 Statistical analyses

We performed all statistical analyses using R (R Version 3.3.2, R Development Core Team, 2016). To analyze the effect of land‐use intensity on ants, we created two generalized linear model (GLM, with Poisson error distribution) with number of ant species as the response variable in the first model (1) and the compound LUI and the region as predictor variables which were allowed to interact and the second model (2) with the three different land‐use components (mowing‐, grazing‐, and fertilization intensity) as predictor variable (for *n* = 110 plots).

To analyze the direct and indirect effects of land use on ants, we used a multimodel inference approach and structural equation models (*SEM*). Since the continuous variables were measured at very different scales, we rescaled them to zero mean and unit variance using the “decostand”‐function of the R package “vegan” (Oksanen, Blanchet, Kindt, Legendre, & O'Hara, 2016). We created a first global model (GM‐1) (LME) with the number of ant species as response variable and the three land‐use components as well as all environmental variables as predictor variables with the region as random factor over all plots (for *n* = 96). Due to missing data, we had to remove 14 plots from this analysis (Alb 8 plots, Hainich 1 plot, Schorfheide 5 plots). To reveal possible effects of the different livestock types on ants, we created a second global model which was similar to the first model but was restricted to only pasture and mown pasture plots (*n* = 61) and included the livestock type to the global model (GM‐2). We used the “dredge” function of the R package “MuMIn” (Bartón, 2016) which generates a set of models with all possible combinations of predictor variables and weighted the models based on their Akaike information criteria for small samples sizes (AICc). We used all models with a ΔAIC_c_ < 2 and applied the “model.avg” function and subsequently the “importance” function which states the relative importance values of each variable calculated as the sum of AICc weights over all models in which the variable appears. Using these variables, we fitted a piecewise structural equation model (piecewise *SEM*) using the R package “piecewiseSEM” (Lefcheck, 2016) to test for direct and indirect effects of the most important variables (all variables selected by model averaging) on the response variable. As suggested in Lefcheck (2016), we standardized the path regression coefficients by scaling them by mean and variance. The structure of both SEMs is described in the appendix. We could not perform the goodness‐of‐fit test (Fisher's C test) for both of our SEMs as they were both fully saturated, with each path being based on a plausible hypothesis.

To analyze the effect of land‐use intensity on the number of sampled ant nests, we created two generalized linear model (GLM, with Poisson error distribution) with number of ant nests as response variable and (1) the compound LUI as predictor variable and (2) with the three different land‐use components (mowing‐, grazing‐, and fertilization intensity) as predictor variables (for *n* = 110 plots for both). In order to study the effect of land‐use intensification on the trait space of morphological and life history traits and to investigate which traits are affected, we performed an ordination analysis. We grouped land‐use intensities into three categories (1/3 of the LUI each—low: LUI: <1.16 (*n* = 29 plots), medium: LUI: 1.17–2.33 (*n* = 69 plots), high: LUI > 2.34 (*n* = 12 plots)). Using the function “gowdis” in the package “FD” (Laliberté, Legendre, Shipley, & Laliberté, 2014; Laliberté & Legendre, 2010), we created a Gower distance matrix form the trait data. Subsequently, a nonmetric multidimensional scaling with two axes on the Gower distance matrix was performed using the “metaMDS” function in the R package “vegan” (Oksanen et al., 2016). The trait data were plotted post hoc with 1,000 permutations using the function “envit.” Differences between the trait space of the LUI categories were tested with a PERMANOVA using “adonis” function with 1,000 permutations (*n* = 110 plots).

## 3 RESULTS

Over all 110 plots, we found 31 ant species belonging to 8 genera. While the regions Alb and Hainich had a similar range of species numbers (Table 1), the region Schorfheide was less diverse. The regions differed in their land‐use intensity. The gradient of land‐use intensity was broader in the Alb and Hainich in comparison with Schorfheide where plots were mainly managed at a medium intensity (Supporting information Figure S1).

### 3.1 Effects of land use on ant species richness

**Figure 1:**
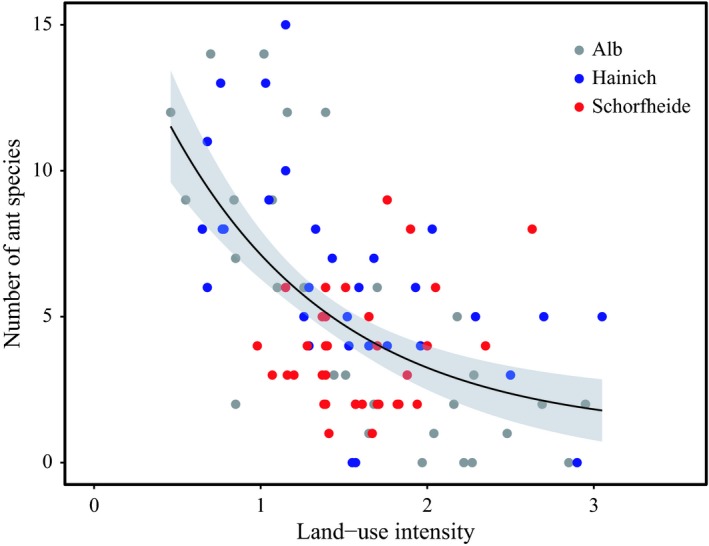
Ant species richness along the land‐use intensity gradient in the three study regions over all plots (*n* = 110). The black line represents the exponential function of a GLM for species number and increasing land‐use intensity. The gray area represents the 95% confidence interval

A high land‐use intensity (LUI) had a significant negative effect on ant species richness, compared over all regions (GLM: χ_1_^2^ = 65.15; *p* < 0.001, Figure 1). Among the regions, similar negative effects were found for the Hainich (*z* = −4.54; *p* < 0.001; Supporting information Figure S1) and Alb (*z* = −7.55; *p* < 0.001; Supporting information Figure S1), but not for the Schorfheide (z = 2.06; *p* = 0.039; Supporting information Figure S1) where we found positive effects. For the three land‐use categories, the effect of mowing was the strongest (negative, GLM: χ_1_^2^ = 68.47; *p* < 0.001; Supporting information Figure S2), followed by grazing (negative, GLM: χ_1_^2^ = 24.05; *p* < 0.001; Supporting information Figure S2). Fertilization had no significant effect (GLM: χ_1_^2^ = 0.17; *p* = 0.68; Supporting information Figure S2).

### 3.2 Effects of environmental variables and land‐use components on ant species richness

**Figure 2:**
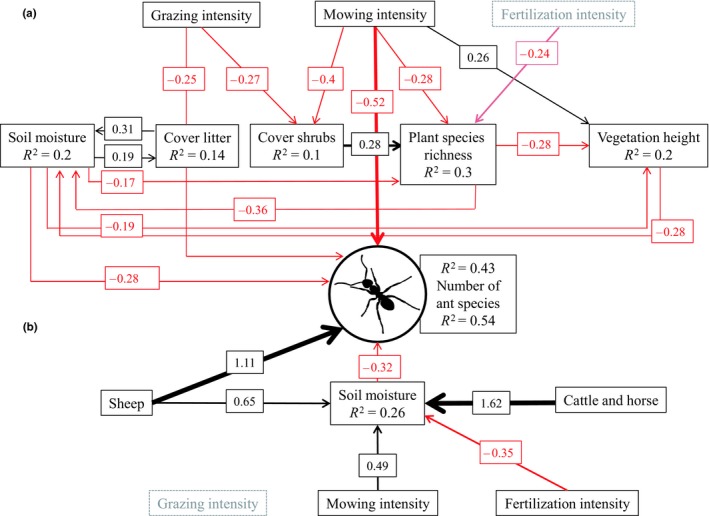
Piecewise structural equation model showing direct and indirect effects of land use and environmental parameters on ant species richness (a) over all sampled plots (*n* = 96) and (b) only on pastures and mown pastures with the livestock type included (*n* = 61). Arrows represent significant (*p* < 0.05) unidirectional interactions between variables (black show positive, red negative interactions). We report the significant path coefficients as standardized effect sizes next to the arrows (all effect sizes are shown in Supporting information Table S5). *R* ^2^ values for component models are given in the boxes of their response variables

The first multimodel averaging approach revealed that seven of the twelve predictor variables (GM‐1) were important for explaining ant species richness (Table 2a). The piecewise *SEM* on the drivers of ant species richness (Figure 2a) showed that a high mowing intensity (β* = *−2.15, standardized coefficient) and a high grazing intensity (β = −0.67, standardized coefficient) had direct negative effects. In addition, a high soil moisture had direct negative effects on ant species richness (β* = *−0.10, standardized coefficient). High mowing and grazing intensities had negative effects on plant species richness.

**Table 2:** Results of the multimodel averaging approach for ant species richness (a) for all plots (*n* = 96) and (b) for all plots with livestock (*n* = 69)

(a) Variable	Importance (%)	N‐containing models	(b) Variable	Importance (%)	N‐containing models
Mowing intensity	100	6	Livestock	100	6
Grazing intensity	100	6	Soil moisture	100	6
Soil moisture	100	6	Mowing intensity	71	4
Plant species richness	67	4	Fertilization intensity	43	3
Vegetation height	61	4	Cover shrubs	27	2
Cover litter	28	2	Grazing intensity	15	1
Cover shrubs	11	1			

The second multimodel averaging approach (GM‐2; only plots with livestock) revealed that six of the thirteen variables used were important for explaining ant species richness (Table 2b). The piecewise *SEM* (Figure 2b) showed the effect of land use including livestock type (compared with cattle as livestock) on ant species richness. In contrast to grazing with cattle, grazing by sheep had significant positive effects on ant species richness (β* = *1.11, standardized coefficient). The mowing and fertilization intensity had indirect effects by influencing soil moisture, which had a negative effect on ant species richness (β *= *−0.13, standardized coefficient).

### 3.3 Effects of land use on ant species compositions

**Figure 3:**
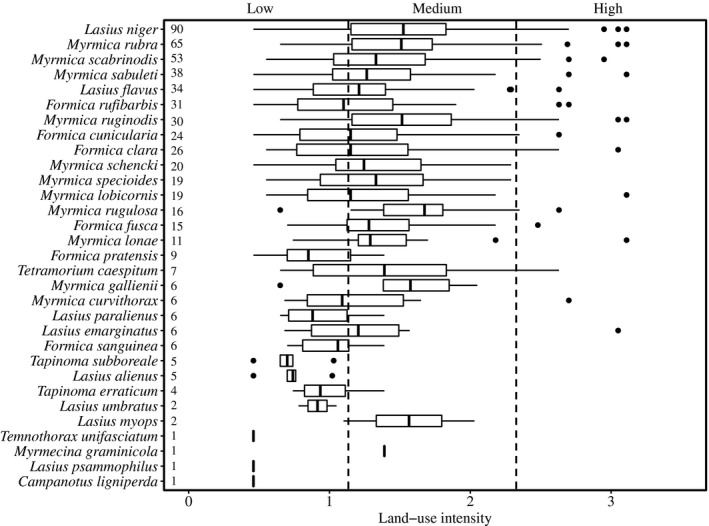
Boxplot showing the occurrences of all ant species present in the three regions along the land‐use intensity gradient, sorted by decreasing number of occurrences. The numbers on the left figure margin state the number of plots where the species occurred. Dashed lines show categories of low, medium, and high land‐use intensity. Black dots indicate outliers

Land‐use intensity strongly impacted the occurrences of the different species (Figure 3). Very common species (based on number of plots where these species occurred on) with high tolerance to different levels of land‐use intensity were, for example, *Lasius niger,* *Myrmica rubra,* and *M. scabrinodis*. Other common species, like *Formica rufibarbis*, *F. cunicularia, *or *F. clara,* were rarely found on plots with high land‐use intensity. Among the less common species, *Myrmica gallienii* or *Tetramorium caespitum* showed higher tolerances to high land‐use intensity than for instance, *Lasius paralienus* or *Formica sanguinea*. The rarest species occurred mostly in low‐intensity managed plots.

**Figure 4:**
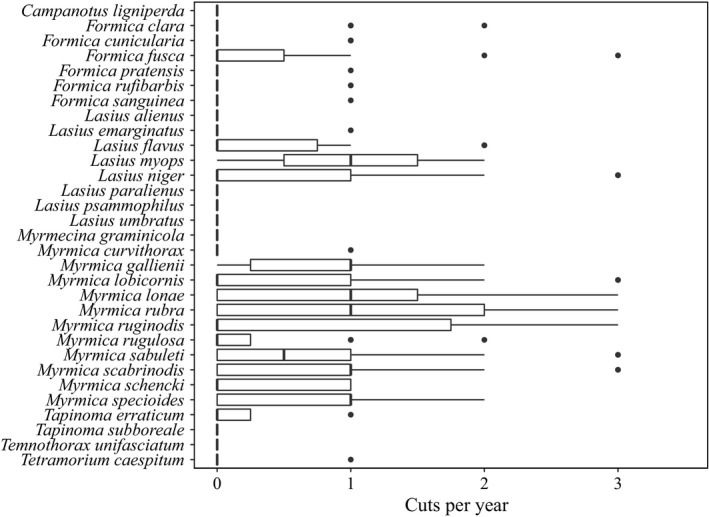
Boxplot showing the occurrence of all ant species present in the three regions along the number of cuts per year, alphabetically ordered. Black dots indicate outliers

Since mowing was the land‐use practice with the strongest negative effect on ant species richness, we additionally plotted the species occurrences along the number of cuts per year. This revealed that most *Formica* species and *Lasius flavus* were highly sensitive toward mowing while *Lasius niger* and most *Myrmica* species tolerated higher mowing intensities (Figure 4).

Ant abundance measured as the number of nests per plot was significantly negatively influenced by increased land‐use (GLM: χ_1_^2^ = 9.29; *p* < 0.002; Supporting information Figures S3, S4). Separately analyzed for the three land‐use categories, we did not find significant effects for mowing (GLM: χ_1_^2^ = 2.32; *p* = 0.13), grazing (GLM: χ_1_^2^ = 0.08; *p* = 0.78), and fertilization (GLM: χ_1_^2^ = 2.29; *p* = 0.13).

**Figure 5:**
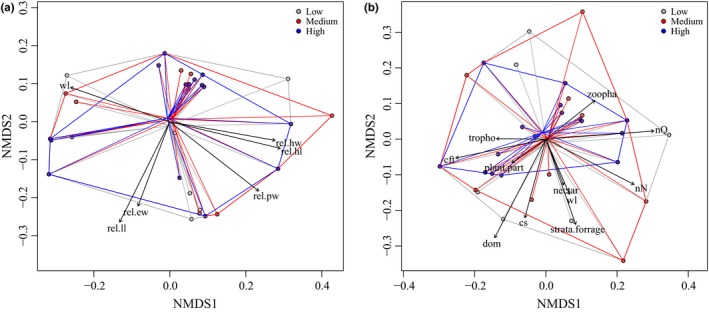
Ordination plot showing the trait space covered by ant species occurring under low, medium, and high land‐use intensity (different colors gray, red, and blue dots, respectively). For each, a nonmetric multidimensional scaling (NMDS) was conducted based on a Gower distance matrix. (a) Plot is based on morphological traits. Used morphological traits are as follows: Webers' length (wl), relative leg length (rel.ll), relative pronotum width (rel.pw), relative head width (rel.hw), relative head length (rel.hl), relative eye width (rel. ew). (b) Plot is based on life history species traits. Used trait values are as follows: strata species is most likely to be found foraging (strata forage), assumed percentage animal diet of total food intake (zoopha), assumed percentage of nectar diet of total food intake (nectar), assumed percentage trophobiosis‐based diet of total food intake (tropho), assumed percentage plant based diet of total food intake (plant), Weber's length (wl), colony size ln transformed (cs), behavioral dominance (dom), number of queens per nest (nQ), number of nests per colony (nN), colony foundation type (cft)

Land‐use intensity also affected the functional trait space of ant communities. Overall, ant community functional trait space of morphological traits (PERMANOVA: *F*_1_ = 0.35, *p* = 0.72, Figure 5a) and life history traits (PERMANOVA: *F*_1_ = 0.34, *p* = 0.78) did not change significantly among plots managed at low, medium, and high land‐use intensities. However, functional trait space for life history traits decreased strongly with increasing management intensity (Figure 5b). At high management intensities, species communities consist of species which foraged less in higher vegetation and had smaller and less polydomous colonies (Figure 5b). The morphological trait space did not differ between land‐use intensities, since several species of the genera *Formica*, *Lasius,* and *Myrmica* occurred occasionally even at high intensities.

## 4 DISCUSSION

We found a decrease in ant species richness, number of ant nests, functional trait space (life history traits), and a change of ant community composition with increasing land‐use intensity. These negative effects were found for the regions Hainich and Alb, but not at the Schorfheide, where sampled grassland sites had a relatively narrow range of management intensities and ant species richness.

### 4.1 Effects of land use on ant species richness

When comparing the three management practices, only mowing and grazing had significant, negative effects on ant species richness. The way in which mowing influences temperate ant communities is not well understood since former studies have focused on the time of mowing (Grill, Cleary, Stettmer, Bräu, & Settele, 2008; Korösi et al., 2014) rather than on mowing intensity. For example, Dahms, Wellstein, Wolters, and Dauber (2005) found no effect of low‐intensity mowing (once or twice per year) compared with other low‐intensity management types (mown pastures, cattle pastures, and silage meadow). Negative effects of intensive mowing were also found on the taxonomic richness of other groups including plants, fungi, arthropods, and vertebrates and overall herbivory (i.e., insects and slugs; Gossner, Weisser, & Meyer, 2014) and caused a decrease of mainly rare species in multiple arthropod taxa (Simons et al., 2015). The tremendous negative effect of mowing on ants becomes particularly clear by looking at the mean number of species per plot. Unmown plots had a mean species richness of 7.1 species, but the number decreased at plots mown once (4.1 species), twice (2.6 species), and three times per year (2.7 species). In our study, we found that ant species which are directly threatened by mowing were mainly species which build aboveground nests such as most *Formica* species and *Lasius flavus*. These species build large nests as “heat collectors” in which the brood is transferred for optimal larval growth (Penick & Tschinkel, 2008). The destruction of these nests through mowing with machines can lead to a significant loss of brood and workers. Further, since many species start their mating flights in June (Seifert, 2007), mowing in late spring or early summer can reduce the number of queens and males dramatically.

Effects of grazing on ants depended on grazing intensity as well as livestock type. High grazing intensity affected ant species richness negatively. However, in the present study pastures grazed at high intensity were only stocked with cattle. In comparison, grazing by sheep or cattle pastures with a low grazing intensity due to a short grazing duration and a low number of livestock showed a significantly higher ant species richness. Our study thus corroborates the findings of other studies where grazing by sheep had positive effects on ant abundance and biomass (Hutchinson & King, 1980) as well as other arthropods (Dennis et al., 2008; Simons et al., 2014; review: Scohier & Dumont, 2012). These positive effects may be mediated by higher habitat heterogeneity (Hoffmann & James, 2011) and a higher number of vascular plant species (Socher et al., 2013) on pastures grazed by sheep and generally pastures grazed at low intensity. Plant species richness is decreased on intensively managed, frequently mown plots which have higher percentages of fast‐growing plant species (Socher et al., 2013), resulting in an increased average vegetation height.

Fertilization intensity only had an indirect effect on ants by altering plant communities. Nitrogen increases the plant growth, favoring especially fast‐growing plant species (Socher et al., 2013), decreasing the sun exposure of the soil surface, and resulting in a higher soil moisture. This had a strong negative effect on ant species richness in our study sites, corroborating previous studies (Dahms et al., 2005; Pérez‐Sánchez et al., 2017; Seifert, 2017).

Temperate ant species are thermophiles and prefer warm and dry habitats (Sanders, Lessard, Fitzpatrick, & Dunn, 2007) with a rather small range of soil moisture tolerated by most species (Seifert, 2017). However, the (ground) temperature was not among the most important variables in our analyses. This can be explained by the low temperature range among the plots (85% of plots had an average ground temperature between 16 and 18°C). Furthermore, the temperature measurement of ten centimeters above the ground might not directly represent the warm temperatures on sun‐exposed ground. But since soil moisture and sun exposure should be correlated with each other, we assume that plots with a high soil moisture also have a lower ground temperature. Indeed, across Central Europe, Seifert (2017) found the highest average number of species in warm grassland with a good soil drainage (see also Dekoninck, Koninck, Baugnée, and Maelfait (2007)) and fewer ant species in more humid meadows or pastures.

Among the three study regions, the grassland plots in Schorfheide had the lowest mean soil moisture. However, overall species richness was relatively low in this region and decreased strongly with increasing soil moisture. Pastures in the Schorfheide were never grazed by sheep, which was where high ant species richness was found in the Alb and Hainich. Recently, Grevé et al. (2018) showed that forest plots at the Schorfheide are rather dry and species‐rich, which implies a potentially large regional species pool. This suggests that a potential increase of ant species richness in grassland plots could be achieved by a change in the grassland management, shifting from cattle to sheep pastures.

### 4.2 Effects of land use on functional trait space and ant community compositions

The functional trait space of ant communities decreased with increasing land‐use intensity. At high land‐use intensities, 14 of 31 species were absent and most others, such as *Formica* species, occurred only occasionally. The most common species on high‐intensity plots were *L. niger*, *M. rubra,* and *M. scabriondis* which are widespread, very common in temperate grasslands, and known to be rather unaffected by grassland management (Dauber & Wolters, 2004, 2005; Grill et al., 2008; Seifert, 2017). Species disappearing first under increasing land‐use intensity are open habitat specialists like *Tapinoma erraticum* and *T. subboreale* or the three species of the *Lasius paralienus*‐complex: *L. alienus*, *L. paralienus,* and *L. psammophilus, *which require warm habitats with reduced plant cover such as nutrient‐poor grasslands or sheep pastures (Seifert, 2007, 2017). Species communities in grasslands managed at high intensities, lacked species forming large and polydomous colonies or foraging higher up in the vegetation. Especially the meadow ant *Formica pratensis*, a key species in temperate European grasslands appears to be highly vulnerable. This species forms large, polydomous nests and is found primarily in grasslands with at least some woody plants (Seifert, 2007). High land‐use intensities, and in particular a high mowing frequency, diminish the required structural and plant diversity in grasslands for such species and additionally lead to the destruction of nests.

Ant species living in intensively used grasslands must be disturbance‐resistant, euryoecious and able to tolerate large climatic variations as the ground temperature increases, and soil humidity declines rapidly after mowing (Seifert & Pannier 2007). Beside *L. niger*, different *Myrmica* species can tolerate these climatic variations. Species of this genus can often tolerate dry and wet conditions and are dietary generalists (Radchenko & Elmes, 2010; Seifert, 2017). *Myrmica *species showed the highest tolerance to mowing, as these species build nests with a rather small nest mound and a larger part of the nest being underground (Radchenko & Elmes, 2010), making them less affected by mowing. However, in contrast to other genera like *Formica* and *Lasius*, *Myrmica* species are rather similar in shape and lifestyle, which results in a low functional trait space.

## 5 CONCLUSIONS

To our knowledge, this is the first study analyzing the effect of land use in grasslands from low to high intensity, covering a large number of grassland plots and disentangling the effect of different management types (mowing, grazing and fertilization). Increasing land‐use intensity reduced the number of ant species and the number of (aboveground) nests. In addition, it led to a reduced functional diversity and caused species community homogenization. Therefore, we can show a similar response of ants to higher land‐use intensities as shown for multiple other groups such as plants, herbivores, secondary and tertiary consumers, and as such, all parts of the trophic pyramid of the grassland ecosystem (Gossner et al., 2016). To preserve species‐rich grassland ant communities, we highly recommend reducing grassland management intensity, especially the number of mowing events per year. A transformation of nutrient‐rich meadows with a low plant species richness and high soil moisture toward a nutrient‐poor pasture with a higher diversity of plant species would be beneficial for ant species. Particularly low management intensities, as provided by sheep grazing, are a suitable method to increase habitat heterogeneity and maintain and fulfill the demands of specialized open habitat ant species.

## CONFLICT OF INTEREST

None declared.

## AUTHOR CONTRIBUTIONS

HF conceived the study. LH did the field wk. LH and MEG identified the ant species. DS and VB provided data and expertise. LH, MG, and HF analyzed the data and wrote the first version of the manuscript. All auths helped to improve the manuscript

## Data Availability

This work is based on data elaborated by several projects of the Biodiversity Exploratories program (DFG Priority Program 1374). Part of the data used are available on the Biodiversity Exploratories Information System (http://doi.org/10.17616/R32P9Q) at https://www.bexis.uni-jena.de/PublicData/PublicData.aspx (IDs: 23986, 1000). However, to give data owners and collectors time to perform their analysis the data and publication policy of the Biodiversity Exploratories include by default an embargo period of three years from the end of data collection/data assembly which is valid for the remaining data (IDs: 19809). These datasets will be made publicly available at the same data repository.
