# Supplementary material for: Direct and indirect effects of land‐use intensification on ant communities in temperate grasslands
Source: Ecol Evol. 2019 Mar 5;9(7):4013–24. doi: 10.1002/ece3.5030 (PMC6468076; doi:10.1002/ece3.5030)
Supplement: Supplementary file 1 [file ECE3-9-4013-s001.pdf]

Direct and indirect effects of land-use intensification on ant communities in temperate grasslands

Lisa Heuss [1+], Michael E. Grevé [1+*], Deborah Schäfer[2], Verena Busch[3], Heike Feldhaar[1]

[1] Animal Population Ecology, Animal Ecology I, Bayreuth Center of Ecology and Environmental Research (BayCEER), University of Bayreuth, Universitätsstr. 30, 95447 Bayreuth, Germany

[2] University of Bern, Institute of Plant Sciences, Altenbergrain 21, 3013 Bern, Switzerland

[3] Münster University, Institute for Landscape Ecology, Heisenbergstr. 2, 48149 Münster, Germany

[+] Authors contributed equally

* Corresponding author: Michael.greve@uni-bayreuth.de

Appendix

Detailed description of the three study areas

The biosphere reserve Schwäbische Alb is located in the low mountain ranges of south-west Germany (48°43'N 9°37'E; 6-7 °C mean annual temperature, 700-1000 mm mean annual rainfall, elevation of 460-860 m a.s.l., and a spatial extent of 420 km^2). The national park of Hainch-Dün and its surrounding areas is located in central Germany (51°20'N 124 10°41'E, 6.5-8°C, 500-800 mm, 285–550 m a.s.l., 1300 km^2). The biosphere reserve Schorfheide-Chorin is situated in the lowlands of north-eastern Germany in a young glacial landscape with many wetlands (53°02'N 13°83'E, 8-8.5 °C, 500-600 mm, 3-140 m a.s.l., 1300 km^2). The study areas have a latitudinal distance of around 600 km between Alb and Schorfheide

Detailed description of pitfall trap sampling

Three pitfalls were placed on each side of the plot at 12.5 m, 25 m, and 37.5 m from the corner. As pitfalls, plastic cups (Ø = 70 mm) were placed in the soil with the opening at surface level and filled with a solution of water, salt, and soap (200 g salt, 1 ml soap per liter water). Pitfalls were collected on the third day after installation.

Detailed description of baiting

Four bait stations were placed on each side at 7,5 m, 17,5 m, 32,5 m and 42,5 m from the corners. As baiting station we used petri dishes (Ø = 90 mm) with a round filter paper and placed five baits made from an artificial diet (based on whey protein, caseinate, egg powder, sucrose, agar, and water, Table S1) on each filter paper that contained different protein to carbohydrate ratios (1:1, 1:2.5, 1:5, 1:7.5 and 1:10; protein:carbohydrate) to attract all ants feeding on different food sources. Baits were comprised of whey and casein protein as protein source and sucrose and carbohydrate source dissolved in water and gelled with agar to produce homogenous cubes of approximately 1 cm^3. We counted all ants at these baiting stations 30 minutes and 60 minutes after placing them in the field.

Table S1. Artificial diet used for bait stations

Propor-tion P:C	Whey protein (g)	Caseinat (g)	Egg powder (g)	Succrose (g)	Vitamines (g)	Benzoat (g)	Agar (g)	Water (ml)
1:1	33.5	30.52	11	60.0	2	1	8	600
1:2.5	17.82	16.24	11	85.71	2	1	8	600
1:5	9.11	8.3	11	100.0	2	1	8	600
1:7.5	5.52	5.03	11	105.88	2	1	8	600
1:10	3.57	3.25	11	109.09	2	1	8	600

Detailed description of SEMs

To analyze all direct and indirect effects of land-use components and environmental variables, which were found to be affecting ant species richness, we used structural equation modeling. First for all sampled plots (n=96) and second for only grazed plots (n=61) to analyze the effects of different livestock types separately. The region was used as random effect. The structure of each linear model within the SEM is: LME (response variable ~ all possible predictor variables added up by +, region as random effect)

Structure of the first SEM over all 96 plots:

SEM1= (

lme (Number of ant species ~ Mowing intensity + Fertilisation intensity + Grazing intensity + Soil moisture + Number of vascular plants + Mean vegetation height (cm) + Cover of litter (%) + Cover of shrubs (%), random = ~ 1 | Region),

lme (Number of vascular plants ~ Mowing intensity + Fertilisation intensity + Grazing intensity + Soil moisture + Cover of litter (%) + Cover of shrubs (%), random = ~ 1 | Region),

lme (Mean vegetation height (cm) ~ Mowing intensity + Fertilisation intensity + Grazing intensity + Soil moisture + Number of vascular plants + Cover of litter (%) + Cover of shrubs (%), random = ~ 1 | Region),

lme (Cover of litter (%) ~ Mowing intensity + Fertilisation intensity + Grazing intensity + Soil moisture + Number of vascular plants + Mean vegetation height (cm) + Cover of shrubs (%), random = ~ 1 | Region),

lme (Soil moisture ~ Mowing intensity + Fertilisation intensity + Grazing intensity +Number of vascular plants + Mean vegetation height (cm)+ Cover of litter (%)+ Cover of shrubs (%), random = ~ 1 | Region),

lme (Cover of shrubs (%) ~ Mowing intensity + Fertilisation intensity + Grazing intensity + Soil moisture, random = ~ 1 | Region))

Structure of the second SEM including only pasture- and mown pasture-plots.

SEM2 = (

lme (Number of ant species ~ Soil moisture + Livestock type + Mowing intensity + Fertilisation intensity + Cover of shrubs (%) + Grazing intensity + pH, random = ~ 1 | Region),

lme (Soil moisture ~ Livestock type + Mowing intensity + Fertilisation intensity + Cover of shrubs (%)+ Grazing intensity, random = ~ 1 | Region),

lme (Cover of shrubs (%)~ Soil moisture + Livestock type + Mowing intensity + Fertilisation intensity + Grazing intensity, random = ~ 1 | Region))

Table S2. Ant species found in Alb, Hanich and Schorfheide with the trait values used for the calculation of FD_{LH}. Trait information marked with * are taken from Seifert (2007, 2017) and marked with + are taken from Arnan et al. (2017). Abbreviations: strata forage, vertical strata where species is most likely to be found foraging (calculation and trait data see Table S3); zoopha, assumed percentage animal diet of total food intake; nectar, assumed percentage of nectar diet of total food intake; tropho, assumed percentage trophobiosis based diet of total food intake; plant, assumed percentage plant based diet of total food intake; WL = Weber's length; CS =colony size ln transformed; Dom = behavioral dominance, nQ = number of queens per nest, nN = number of nests per colony; CFT= colony foundation type. Data type and additional information on the traits are provided in Table S3.

Species	Zoopha*	Nectar*	Tropho*	Plant part*	WL	Dom+*	CS+*	nQ+*	nN+*	CFT+*	Strata forage*
Campanotus ligniperda	0.27	0.05	0.63	0.05	3.83	1	7.82	0.5	0	1	1.99
Formica clara	0.6	0.05	0.29	0.06	2.26	1	8.52	0	0	1	0.93
Formica cunicularia	0.58	0.05	0.32	0.05	1.92	0	7.24	0	0.5	1	1.05
Formica fusca	0.5	0.1	0.35	0.05	1.95	0	9.1	1	1	1	1.08
Formica pratensis	0.14	0.02	0.55	0.02	2.71	1	11	1	1	0	0.97
Formica rufibarbis	0.59	0.05	0.31	0.05	2.27	0	6.91	0	0	1	1.04
Formica sanguinea	0.48	0.02	0.48	0.02	2.61	1	9.21	0.5	0	0.5	1.24
Lasius alienus	0.39	0.18	0.37	0.06	1.15	1	9.47	0	0	1	0.76
Lasius emarginatus	0.45	0.05	0.44	0.06	1.32	1	9.21	0	0	1	1.49
Lasius flavus	0.2	0	0.8	0	1.32	0	9.21	0.5	0	1	-2.08
Lasius myops	0.2	0	0.8	0	0.85	0	8.52	0	0	1	-2.08
Lasius niger	0.34	0.05	0.56	0.05	1.23	1	9.21	0	0	1	1.05
Lasius paralienus	0.39	0.19	0.36	0.06	1.11	1	9.21	0	0	1	0.76
Lasius psammophilus	0.35	0.14	0.5	0.01	1.0	0	10.43	0	1	1	0.53
Lasius umbratus	0.2	0	0.8	0	1.32	0	8.01	0.5	0	0	-2.08
Myrmecina graminicola	0.99	0	0	0.01	0.95	0	4.61	0.5	0	0	-1.36
Myrmica curvithorax	0.56	0.04	0.35	0.05	1.45	1	6.68	0	0	1	0.12
Myrmica gallienii	0.6	0.06	0.32	0.02	1.61	1	6.72	1	0	1	0.27
Myrmica lobicornis	0.72	0.04	0.2	0.04	1.53	0	5.63	0.5	0	0.5	-0.26
Myrmica lonae	0.54	0.04	0.35	0.07	1.51	0	8.01	1	0	0.5	0.42
Myrmica rubra	0.48	0.05	0.37	0.1	1.51	0	8.01	1	1	0.5	0.66
Myrmica ruginodis	0.53	0.02	0.3	0.15	1.69	0	7.6	0.5	0	0.5	0.77
Myrmica rugulosa	0.64	0.08	0.25	0.03	1.45	0	7.6	1	1	0.5	0.13
Myrmica sabuleti	0.51	0.07	0.37	0.05	1.46	0	8.01	1	0	0.5	0.54
Myrmica scabrinodis	0.51	0.06	0.4	0.03	1.43	0	7.31	0.5	0	0.5	0.34

Myrmica schenki	0.58	0.1	0.27	0.05	1.57	0	5.87	0.5	0	0.5	0.29
Myrmica specioides	0.51	0.05	0.41	0.03	1.37	0	7.31	1	0	0.5	0.22
Tapinoma erraticum	0.6	0.13	0.2	0.07	0.96	1	8.16	1	1	0	0.95
Tapinoma subboreale	0.6	0.13	0.2	0.07	0.88	0	NA	1	1	0	0.95
Temnothorax unifasciatum	0.8	0.15	0.05	0	0.72	0	5.78	0	0	1	0.8
Tetramorium caespitum	0.26	0.04	0.35	0.35	0.94	1	9.21	0	0	1	-0.73

Table S3. Description of the different traits for the calculation of the life history trait analyses

Trait	Data type	States
CS	Continuous	Mean colony size (log transformed)
WL	Continuous	Mean Weber´s length of worker (mm)
Zoo, Nectar, Troph, and Plant	Continuous	Assumed relative percentage of animal-, nectar-, trophobiosis- or plant-based diet. All summed to 100 %. Values are partly assumed by Seifert (2017), but also based on very detailed food analyses*
Dom	Binary	(0) Subordinate; (1) Dominant
nQ	Ordinal	(0) Monogyny; (0.5) Monogyny or polygyny; (1) Polygyny
nN	Ordinal	(0) Monodomy; (0.5) Monodomy or polydomy; (1) Polydomy
CFT	Ordinal	(0) Dependent colony founding; (0.5) Dependent and independent colony founding; (1) Independent colony founding
Strata.forage	Continuous	Positive values for higher probability of species found foraging above ground, negative values for foraging under the surface.

Arnan, X., Cerdá, X., & Retana, J. (2017). Relationships among taxonomic, functional, and phylogenetic ant diversity across the biogeographic regions of Europe. *Ecography*, *40*(3), 448–457. https://doi.org/10.1111/ecog.01938

Seifert, B. (2017). The ecology of Central European non-arboreal ants – 37 years of a broad-spectrum analysis under permanent taxonomic control. *Soil Organisms*, *89*(April), 1–67.

Table S4. Foraging strata calculated as the sum of the probability of worker to forage in different vertical strata, where the value for each strata is multiplied by a specific factor, based on predictions of Seifert (2017). Deep soil - foraging in deeper soil at depths of at least 10 cm; top soil - foraging in top soil including the root layer of herbs in depths < 10 cm; moss litter- foraging in the moss and litter layer; free surf = foraging on free, above-ground surfaces except of those on herbs, shrubs and trees; herb = foraging in the herb layer; shrub tree =foraging on shrubs and trees, higher than 2 meters.

Species	Strata.forage	deep soil (multiplied by -3)	top soil (multiplied by -2)	moss litter (multiplied by -1)	free surf (multiplied by 1)	herb (multiplied by 2)	shrub tree (multiplied by 3)
Campanotus ligniperda	1.99	0.03	0.06	0.01	0.21	0.07	0.62
Formica clara	0.93	0.01	0.09	0.03	0.6	0.24	0.03
Formica cunicularia	1.05	0.01	0.11	0.02	0.45	0.36	0.05
Formica fusca	1.08	0.01	0.14	0.05	0.33	0.3	0.17
Formica pratensis	0.97	0.01	0.15	0.03	0.45	0.2	0.16
Formica rufibarbis	1.04	0.01	0.1	0.02	0.5	0.32	0.05
Formica sanguinea	1.24	0.01	0.1	0.02	0.53	0.09	0.26
Lasius alienus	0.76	0.03	0.22	0.04	0.31	0.18	0.22
Lasius emarginatus	1.49	0.01	0.07	0.03	0.4	0.18	0.31
Lasius flavus	-2.08	0.16	0.78	0.05	0.01	0	0
Lasius myops	-2.08	0.16	0.78	0.05	0.01	0	0
Lasius niger	1.05	0.02	0.16	0.04	0.34	0.19	0.25
Lasius paralienus	0.76	0.03	0.22	0.04	0.31	0.18	0.22
Lasius psammophilus	0.53	0.04	0.29	0.04	0.21	0.2	0.22
Lasius umbratus	-2.08	0.16	0.78	0.05	0.01	0	0
Myrmecina graminicola	-1.36	0.05	0.36	0.54	0.05	0	0
Myrmica curvithorax	0.12	0.02	0.25	0.1	0.48	0.15	0
Myrmica gallienii	0.27	0.01	0.18	0.23	0.32	0.21	0.05
Myrmica lobicornis	-0.26	0.02	0.15	0.39	0.39	0.05	0
Myrmica lonae	0.42	0.02	0.1	0.26	0.37	0.18	0.07
Myrmica rubra	0.66	0.02	0.15	0.19	0.26	0.19	0.19
Myrmica ruginodis	0.77	0.01	0.15	0.18	0.26	0.18	0.22
Myrmica rugulosa	0.13	0.02	0.17	0.19	0.52	0.1	0
Myrmica sabuleti	0.54	0.02	0.1	0.21	0.4	0.2	0.07
Myrmica scabrinodis	0.34	0.02	0.18	0.15	0.42	0.2	0.03
Myrmica schencki	0.29	0.02	0.15	0.2	0.41	0.22	0
Myrmica specioides	0.22	0.02	0.18	0.18	0.42	0.2	0
Tapinoma erraticum	0.95	0	0.09	0.1	0.4	0.4	0.01
Tapinoma subboreale	0.95	0	0.09	0.1	0.4	0.4	0.01
Temnothorax unifasciatum	0.8	0	0.06	0.24	0.24	0.46	0
Tetramorium caespitum	-0.73	0.17	0.34	0.03	0.43	0.03	0

Table S5. Path coefficients (and standard error (SE)) of all unidirectional relationships among variables of both piecewise SEM, shown in Fig. 2a and 2b. Significance: p< 0.05: *; p< 0.01: ** p< 0.001: ***

Response	Predictor	Coefficient	SE	P-value	Significance
First SEM over all 96 plots:					
Number of ant species	Mowing intensity	-0.52	0.12	< 0.001	***
	Soil moisture	-0.28	0.08	0.002	**
	Grazing intensity	-0.25	0.10	0.012	*
	Vegetation height	-0.18	0.11	0.088	
	Cover litter	0.14	0.11	0.208	
	Cover shrubs	0.08	0.08	0.349	
	Plant species richness	0.10	0.11	0.390	
	Fertilization intensity	-0.06	0.10	0.514	
Plant species richness	Cover shrubs	0.28	0.07	< 0.001	***
	Fertilization intensity	-0.24	0.09	0.009	**
	Mowing intensity	-0.28	0.11	0.013	*
	Soil moisture	-0.17	0.08	0.031	*
	Grazing intensity	-0.17	0.09	0.063	
	Cover litter	-0.03	0.10	0.736	
Vegetation height	Plant species richness	-0.28	0.11	0.012	*
	Soil moisture	-0.19	0.08	0.026	*
	Mowing intensity	0.26	0.12	0.031	*
	Cover litter	0.17	0.11	0.118	
	Cover shrubs	-0.08	0.08	0.322	
	Grazing intensity	0.06	0.10	0.543	
	Fertilization intensity	0.00	0.10	0.994	
Cover litter	Soil moisture	0.19	0.08	0.021	*
	Vegetation height	0.16	0.10	0.117	
	Fertilization intensity	-0.14	0.09	0.128	
	Grazing intensity	-0.14	0.09	0.141	
	Mowing intensity	0.14	0.12	0.222	
	Cover shrubs	0.04	0.08	0.628	
	Plant species richness	0.01	0.11	0.900	
Soil Moisture	Plant species richness	-0.36	0.14	0.011	*
	Cover litter	0.31	0.13	0.023	*
	Vegetation height	-0.28	0.13	0.035	*
	Fertilization intensity	-0.17	0.12	0.165	
	Grazing intensity	-0.16	0.12	0.201	
	Cover shrubs	0.05	0.11	0.635	
	Mowing intensity	0.07	0.15	0.664	
Cover shrubs	Mowing intensity	-0.40	0.15	0.007	**
	Grazing intensity	-0.27	0.12	0.036	*
	Soil moisture	0.02	0.11	0.818	
	Fertilization intensity	0.02	0.12	0.882	
Second SEM including only pasture- and mown plots:					
Number of ant species	Sheep	1.11	0.25	< 0.001	***
	Soil moisture	-0.32	0.11	0.004	**
	Mowing intensity	-0.23	0.14	0.111	
	Cover shrubs	0.11	0.09	0.243	
	Fertilization intensity	-0.10	0.12	0.372	

	Grazing intensity	-0.09	0.11	0.427	
	Cattle and horses	-0.03	0.58	0.961	
Soil Moisture	Mowing intensity	0.49	0.17	0.006	**
	Fertilization intensity	-0.35	0.14	0.019	*
	Cattle and horses	1.62	0.71	0.027	*
	Sheep	0.65	0.31	0.042	*
	Cover shrubs	0.08	0.12	0.536	
	Grazing intensity	-0.01	0.15	0.969	
Cover shrubs	Sheep	0.38	0.32	0.245	
	Mowing intensity	-0.22	0.19	0.249	
	Grazing intensity	-0.17	0.16	0.304	
	Soil moisture	0.10	0.15	0.502	
	Cattle and horses	-0.08	0.80	0.924	
	Fertilization intensity	0.00	0.17	0.985	

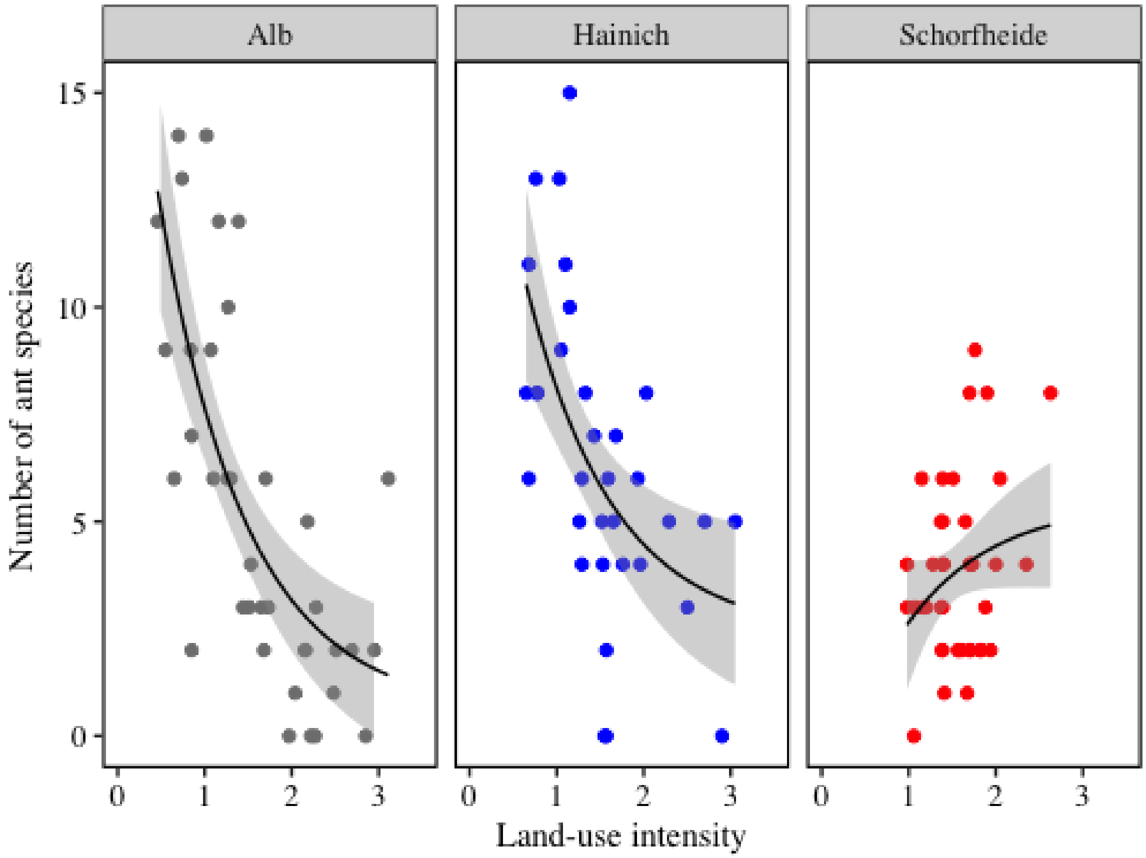

Fig. S1. Number of collected ant species along the land-use gradient for each of the three study regions.The black lines represent the exponential function of a GLM for species number and increasing land-use intensity. The grey areas represent the 95 % confidence intervals.

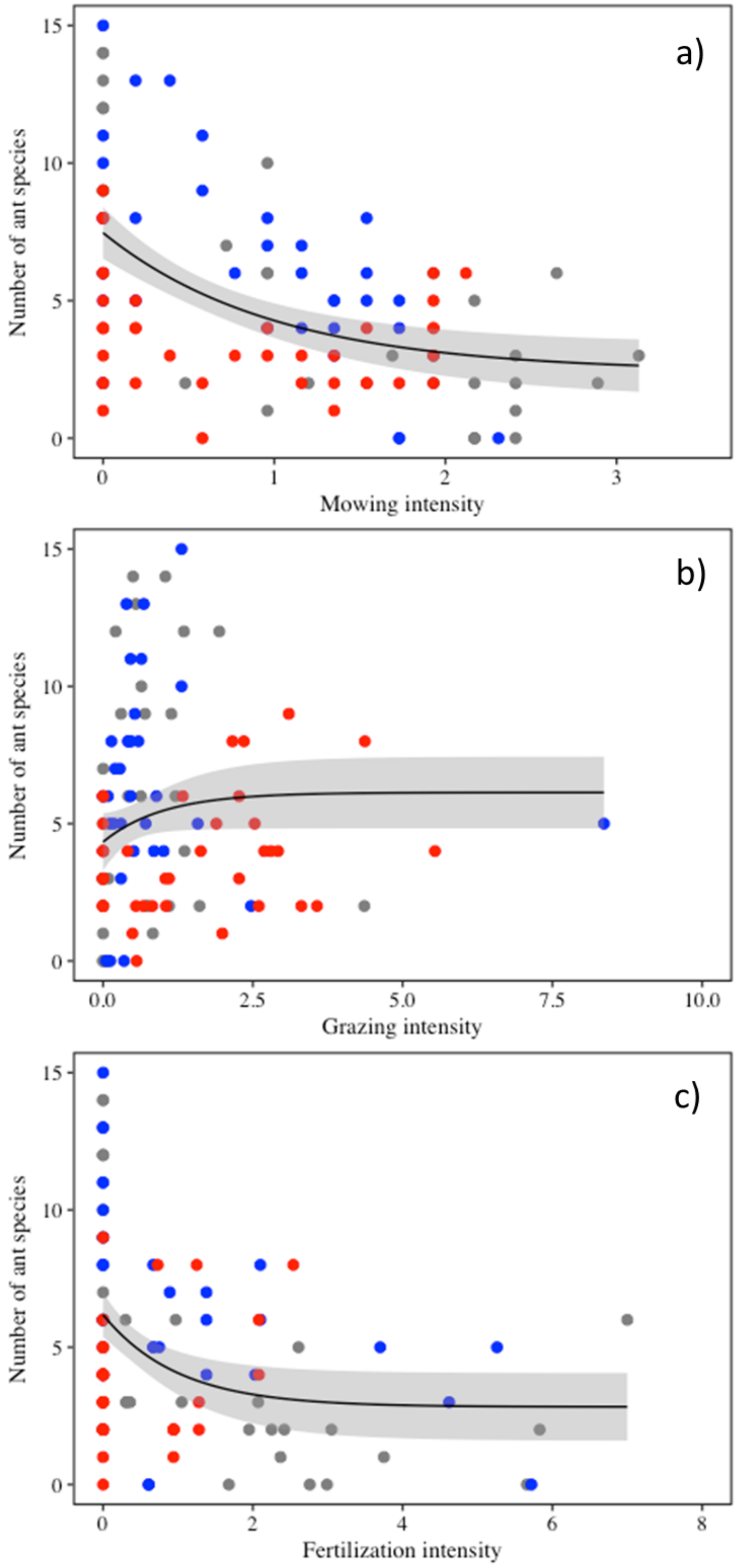

Fig. S2. Effects of mowing (a), grazing (b) and fertilization (c) intensity on number of ant species, grey for Alb, blue for Hainich and red for Schorfheide. The black line represents the exponential function of a GLM for species number and the land-use type. The grey areas represent the 95 % confidence intervals.

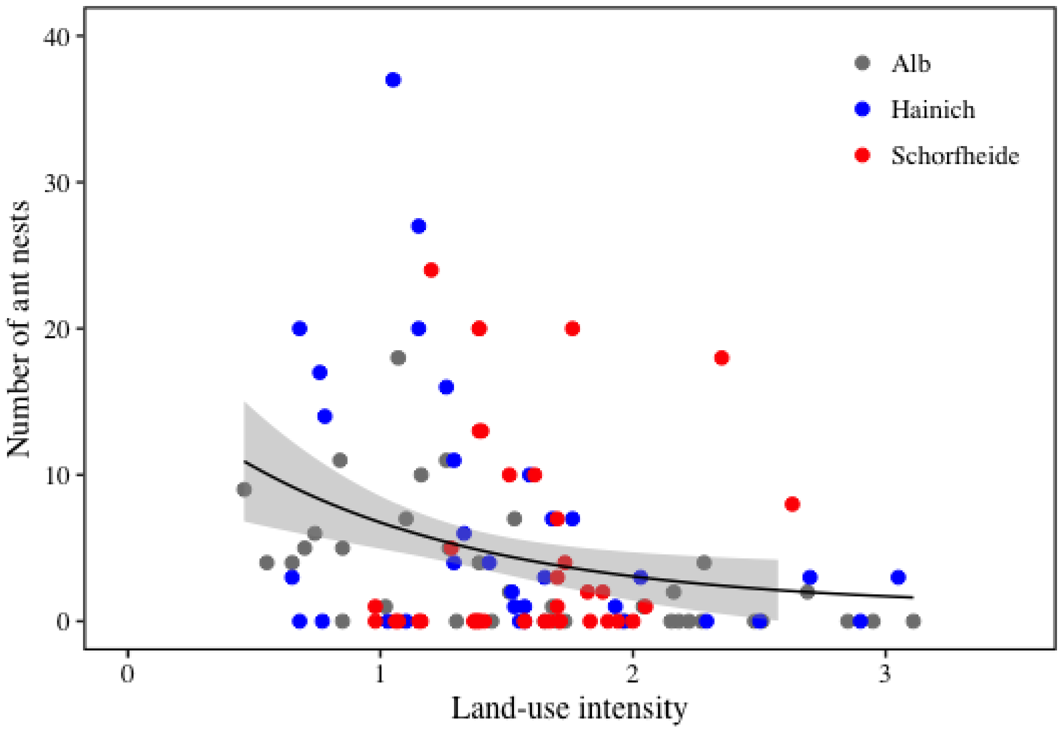

Fig. S3. The number of ant nests (in total 503 nests on 62 plots) in dependence of land-use intensity between the different regions over all plots (n= 110), The black line represents the exponential function of a GLM for number of ant nests and increasing land-use intensity. The grey area represents the 95 % confidence interval.

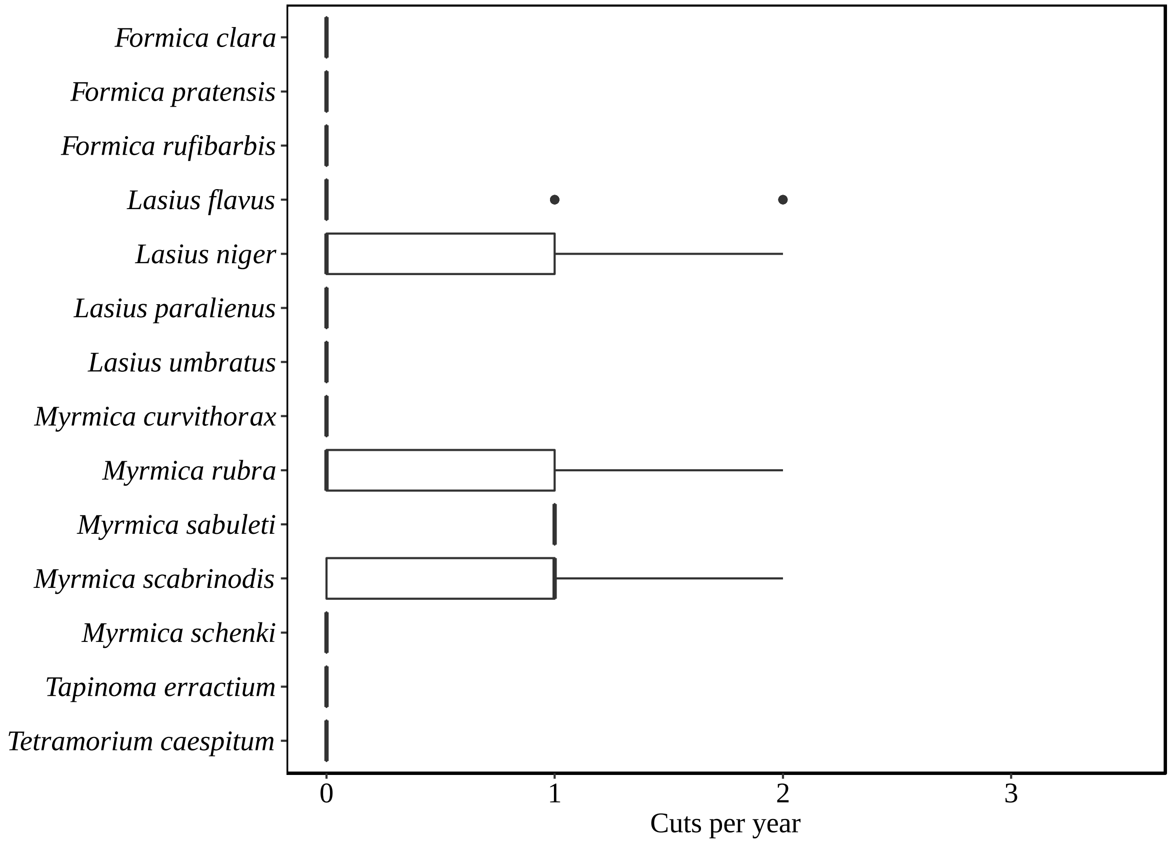

Fig. S4. Boxplot showing the occurrence of visually detected ant nests per species in the three regions along the number of cuts per year, alphabetically ordered. Black dots represent outliers.
